# Modern Sociology

**Published:** 1902-02-22

**Authors:** 


### Modern Sociology.

### SPECIAL SCHOOLS UNDER THE LONDON SCHOOL BOARD.

### II.—For the Deaf.

A generation ago, to be "deaf and dumb" was a calamity from which no escape was possible; for no communication could be held with the speaking and hearing world except through the medium of signs, which must necessarily be known to those addressed by the "deaf-mute;" the range of occupations was restricted, and in the majority of cases, life was a narrow one at the best. Now, however, with the growth of the oral system of teaching, it seems probable that deaf people will soon be able to take their places side by side with the hearing; shut off, it is true, from many pleasures, but not of necessity handicapped to any serious extent as wage-earners or useful members of society. The first class for teaching deaf children under the School Board was at Wilmot Street School, Bethnal Green, where at first four or five children only attended. At this time the question of rival systems of teaching was coming to the front in England; eventually, however, the oral system as practised in Germany was instituted, and since 1880 there has been practically no change in methods; it is used in all the centres, of which there are nineteen, in the London School Board District.

Possibly as time goes on a greater degree of classification will be found necessary, at present the only distinction is that made between children who have never heard at all, and those who, having lost their hearing at a very early age (a frequent cause being neglect after scarlet fever) have a dim recollection of people talking round them. In all there is a tendency to a Gregorian-chant quality of tone, but it is more marked in the case of those born deaf, whose voices have a very archaic sound to the unaccustomed ear. In the case of several children, however, we have been struck with the gentle, pleasant, and refined voice, with a slightly foreign accent, and no trace of cockneyism (thanks to the teacher) or other disagreeable accent; and one is almost inclined to think deafness an advantage in such a case. We have only seen one child whose voice was absolutely unpleasant, so much so that his own mother took every opportunity of sending him away for holidays; and he was so obviously delighted with his own performance that the effect was comic. Some teachers make a point of speaking slowly and distinctly, especially with the beginners; but at one of the centres we have visited, the head teacher was very emphatic on this point, and even with the very little ones spoke herself, and asked us to speak, at the ordinary rate; it was so much better for them, she said, to begin at once to get accustomed to normal speech. This is the whole object of the training, from first to last; and that it is the right line on which to work becomes evident when the boy or girl leaves school, and enters, let us say, upon an apprenticeship to a trade. If the child has not been trained to understand ordinary people, the difficulties are very great, for it can hardly be expected that the foreman or mistress will be as patient as the teacher has been, or will take such pains to make every word intelligible.

Perhaps few people realise what difficulties our mother tongue presents to the deaf child, who has to begin at the very beginning, and learn English as a foreigner does, but with even greater drawbacks. He has to learn the distinction, for example, between the verbs *look* and *see*; and in one clever boy's exercise book we read such a sentence as "After this I went and looked at Esther Smith," meaning that he had been to see her. In an elementary class we visited, there had been a tea-party in honour of the birthday of one of the children; it had formed the subject of a lesson, and on the blackboard were sentences relating to it. But our question, "Who had a party?" completely puzzled the class, merely because the word *who* had not formed part of the lesson, and had not yet been learnt.

The system begins with articulation, and the phonetic value of the vowel sounds. The teacher, in the baby class we first visited, illustrated this by calling a little boy who had only been to school two days, to her side. Taking his hand she breathed the aspirate; he felt her warm breath, and observed the shape of her mouth, and soon he imitated the action, in order to produce the same effect on his own hand. This would be repeated over and over, until what Sir Isaac Pitman calls *hay* was perfectly learnt. The rolling of the R, such a source of grief to our French teachers, was quite an exciting performance at this school. "R-r-r-r!" cried the teacher, pointing first to her own throat and then to the children's, to show where the sound came from, so to speak, and the circle closed round her, each child anxious to be first, if not loudest. Next came simple words, such as "shoe," and here it was evident that a variety of opinion existed, for one little girl, pointing to her own tanned boots, had to have her attention directed to her neighbour's shoes. When the word and the idea were grasped, each child wrote "shoe" on the blackboard. This initial stage being passed, simple sentences are invented, as far as possible with the accompanying action; and later still, advanced lessons in geography, history, arithmetic, or, indeed, any subject capable of comprehension by the elementary scholar's mind, can be taught; and we have heard one little girl answer quite correctly and with almost more than normal intelligence, questions relating to principal, interest, and so forth, in connection with the Bank of England.

At the Balham centre the experiment of boarding-out, so as to get fuller and earlier control over the pupils—some of them from country districts—is being tried with every success; it is found that the children improve both physically and mentally, and the teachers' labour is thereby greatly lessened. At all centres the Board requires a minimum of four and a half hours a week for manual training; and for this the boys attend the carpenters' shop, and the girls the cookery or laundry centre. Otherwise the classes are mixed, for the most part boys and girls learning side by side according to capacity. The attendance, we may remark, is higher than in the ordinary schools. A great need is now being realised for a technical intermediate class, where, after leaving school at 16 (the age fixed by the Board), technical terms could be learnt, so that the first entry into business life might be made less difficult. The Board gives every inducement to parents to send their children young, and though seven is the age fixed, it is as well that they should begin as early as five at least. Learning is no hardship, and these two additional years are of very great value. Much may be done by parents to encourage lip-reading at home and to discourage "monkey tricks," as the teachers call them.

A few deaf children are mentally deficient as well; but in the great majority of cases intelligence seems to be quite up to the normal standard, and the Board of Education, in the special handbook prepared for the Paris Exhibition, says that "the future of deaf-mute education is full of promise." It is encouraging to the teachers to know that one boy at least has recently passed an examination of the College of Preceptors, with exceptionally high marks in algebra, and the two valuable scholarships given by the London County Council will do much to stimulate them to renewed efforts.

